# A comparison of biological characteristics of three strains of Chinese sacbrood virus in *Apis cerana*

**DOI:** 10.1038/srep37424

**Published:** 2016-11-17

**Authors:** Ying Hu, Dongliang Fei, Lili Jiang, Dong Wei, Fangbing Li, Qingyun Diao, Mingxiao Ma

**Affiliations:** 1 Institute of Animal Husbandry and Veterinary, Jinzhou Medical University, Jinzhou, China; 2 Honeybee Research Institute, the Chinese Academy of Agricultural Sciences, Beijing, China

## Abstract

We selected and sequenced the entire genomes of three strains of Chinese sacbrood virus (CSBV): LNQY-2008 (isolated in Qingyuan, Liaoning Province), SXYL-2015 (isolated in Yulin, Shanxi Province), and JLCBS-2014 (isolated in Changbaishan, Jilin Province), by VP1 amino acid (aa) analysis. These strains are endemic in China and infect *Apis cerana*. Nucleotide sequences, deduced amino acid sequences, genetic backgrounds, and other molecular biological characteristics were analysed. We also examined sensitivity of these virus strains to temperature, pH, and organic solvents, as well as to other physicochemical properties. On the basis of these observations, we compared pathogenicity and tested cross-immunogenicity and protective immunity, using antisera raised against each of the three strains. Our results showed that compared with SXYL-2015, LNQY-2008 has a 10-aa deletion and 3-aa deletion (positions 282–291 and 299–301, respectively), whereas JLCBS-2014 has a 17-aa deletion (positions 284–300). However, the three strains showed no obvious differences in physicochemical properties or pathogenicity. Moreover, there was immune cross-reactivity among the antisera raised against the different strains, implying good protective effects of such antisera. The present study should significantly advance the understanding of the pathogenesis of Chinese sacbrood disease, and offers insights into comprehensive prevention and treatment of, as well as possible protection from, the disease by means of an antiserum.

Chinese sacbrood disease (CSD) is a viral disease in honeybees and is caused by sacbrood virus (SBV). SBV is characterised by its ability to spread rapidly and widely^1^,^2^. Since its first identification in *Apis mellifera L.* in the United States in 1913, SBV infection has been found in almost all honeybee colonies throughout the world^3^,^4^,^5^,^6^. SBV mainly infects 2-day-old larvae^7^ and leads to death. Although SBV can also infect adult bees, signs of the disease in them have not been observed yet^8^. Viral infection of *Apis cerana*, the eastern honeybee, was first observed in Guangdong, China, in 1972, and the causative agent was named Chinese sacbrood virus (CSBV). Epidemic outbreaks of the disease occurred in 1972 and 2008 in Liaoning, China, causing death of individual bees and collapse of entire colonies. Since then, the virus has frequently infected *A. cerana* in this region of China, thus dealing a devastating blow to the region’s apiculture.

The genomic organisation of SBV and CSBV resembles that of a typical picornavirus, which is an icosahedral, non-enveloped viral particle 26–30 nm in diameter and belongs to the genus *Iflavirus* in the family Iflaviridae^9^,^10^,^11^. The genome contains one large open reading frame (ORF); the ORFs of SBV and CSBV encode three and four structural proteins, respectively. Ma *et al.* predicted and identified four major CSBV structural proteins by means of bioinformatics and mass spectrometry^12^. There are reported differences between CSBV and SBV in their physicochemical characteristics, pathogenicity, and immunogenicity^13^,^14^,^15^; however, it is unknown whether these differences exist between CSBVs of different genotypes.

In this study, by VP1 amino acid (aa) analysis, CSBVs were subdivided into three divergent groups (I, II, and III), and from each group, strains LNQY-2008, SXYL-2015, and JLCBS-2014, respectively, were selected for analysis of their physicochemical characteristics and pathogenicity. We also investigated antiserum cross-reactivity and protective immunity resulting from immunisation with these strains.

## Results

### Screening of representative strains by multiple sequence alignments of VP1

Sixteen CSBV *VP1* genes of isolates were sequenced (Table 1) and seven were retrieved from GenBank. The length of the *VP1* gene from isolates LNQY-2008, LNQY-2012, JLCC-2011, LNND-2011, LNBX-2009, LNDD-2015, LNJZ-2015, and FZ-2012 (GenBank accession No. KM495267) was found to be 945 nucleotides; in SXYL-2015, HBQHD-2012, LNSZ-2011, BJ-2012 (GenBank: KF960044), SXnor1-2012 (GenBank: KJ000692), GZ-2000 (GenBank: AF251124), and GZ-2002 (GenBank: AF469603), it is 984 nucleotides; and in JLCBS-2014, GZGY-2015, JXJJ-2015, JXNC-2013 (GenBank: KM232611), LNQY-2015, BJ-2015, CQ-2012 (GenBank: KC285046), and SXXA-2015, it is 933 nucleotides.

**Figure 1:**
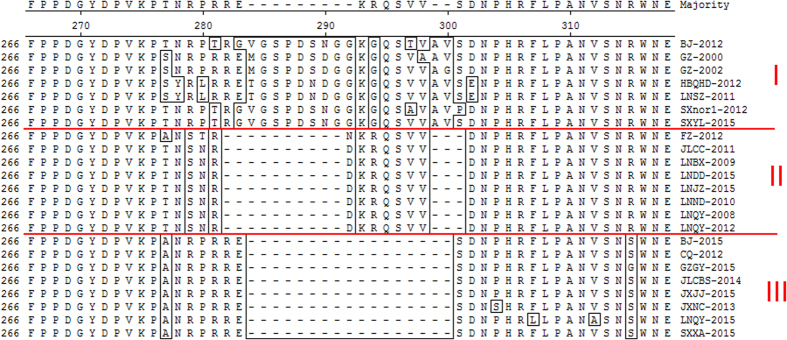
Alignment of all CSBV *VP1* sequences. Based on *VP1* as a target gene, multiple sequence comparisons were carried out for all the CSBV isolates and reference strains from GenBank. In comparison with group I, group II had a 10-aa deletion and 3-aa deletion (positions 282–291 and 299–301, respectively), whereas group III had a 17-aa deletion (positions 284–300).

A multiple alignment of deduced amino acid sequences revealed that the sequences of LNQY-2008, LNQY-2012, JLCC-2011, LNND-2011, LNBX-2009, LNDD-2015, LNJZ-2015, and FZ-2012 are missing 10 aa between amino acid positions 282 and 291 and 3 aa between positions 299 and 301 (compared to SXYL-2015, HBQHD-2012, LNSZ-2011, BJ-2012, SXnor1-2012, GZ-2000, and GZ-2002). Similar comparisons with JLCBS-2014, GZGY-2015, JXJJ-2015, JXNC-2013, LNQY-2015, BJ-2015, CQ-2012, and SXXA-2015 identified a 17-aa deletion corresponding to amino acid positions 284–300 (as compared to SXYL-2015, HBQHD-2012, LNSZ-2011, BJ-2012, SXnor1-2012, GZ-2000, and GZ-2002; Fig. 1).

According to the above analysis, the CSBVs were subdivided into three divergent groups: group I (including SXYL-2015, HBQHD-2012, LNSZ-2011, BJ-2012, SXnor1-2012, GZ-2000, and GZ-2002), group II (including LNQY-2008, LNQY-2012, JLCC-2011, LNND-2011, LNBX-2009, LNDD-2015, LNJZ-2015, and FZ-2012), and group III (including JLCBS-2014, GZGY-2015, JXJJ-2015, JXNC-2013, LNQY-2015, BJ-2015, CQ-2012, and SXXA-2015; Fig. 1). Group I did not have amino acid deletions and was less mutated; therefore, we used the latest isolate SXYL-2015 as a representative strain; the other strains have not been isolated in the same area since 2012 (Table 1). In group II, the amino acid sequence of strain FZ-2012 at positions 87, 192, 195, 202, 242, and 277 was mutated from G, Y, A, Q, K, and T to C, N, V, H, Q, and A, respectively, whereas the other strains are highly conserved. Thus, we used the first isolated and less mutated LNQY-2008 as a representative strain, whose VP1 homology with that of other strains is more than 99.4%, and which reappeared in Qingyuan Liaoning in 2009 and 2010, and in Tieling, Liaoning; Siping, Jilin in 2010 (Table 1). In group III, the strains showed less variation; accordingly, we used JLCBS-2014 (first isolated by our laboratory), which shows less variation as a representative strain, whose VP1 homology with other strains is more than 98.1%, and which reappeared in Changbaishan, Jilin in 2015 (Table 1).

### Analysis of molecular biological characteristics

The complete genome sequences of the three CSBV strains were determined and deposited in GenBank under the following accession numbers: HM237361 for strain LNQY-2008, KU574662 for SXYL-2015, and KU574661 for JLCBS-2014.

The nucleotide sequences of genomes of LNQY-2008, SXYL-2015, and JLCBS-2014 comprise 8863, 8776, and 8794 bp, respectively. The base composition of LNQY-2008 was found to be A (29.63%), G (24.66%), C (16.25%), and U (29.46%), and that of SXYL-2015 was A (29.88%), C (16.53%), G (24.51%), and U (29.08%). The JLCBS-2014 genome was more enriched in A (29.91%) and U (29.24%) than in G (24.35%) and C (16.50%). The LNQY-2008, SXYL-2015, and JLCBS-2014 genomes contain a single, large ORF encoding 2847 aa (starting at nucleotide position 178 and ending at nucleotide position 8721), 2859 aa (starting at nucleotide position 177 and ending at nucleotide position 8756), and 2842 aa (starting at nucleotide position 189 and ending at nucleotide position 8717), respectively. Multiple sequence comparisons of the selected SBV strains, *viz.*, LNQY-2008, SXYL-2015, JLCBS-2014, GZ-2002, BJ-2012, FZ-2012, SXnor1-2012, AcSBV-Kor (GenBank: HQ322114), AmSBV-Kor21 (GenBank: JQ390591), AcSBV-Viet1 (GenBank: KM884990), AcSBV-IndK1A (GenBank: JX270796), and SBV-UK (GenBank: AF092924.1) showed that LNQY-2008 and SXYL-2015 share 90.3–93.7% and 89.8–96.9% nucleotide identity, respectively, with the other SBV isolates. However, JLCBS-2014 showed 89.9–96.2% homology with the other SBV isolates at the nucleotide level (Table 2, Row 1–3).

**Figure 2:**
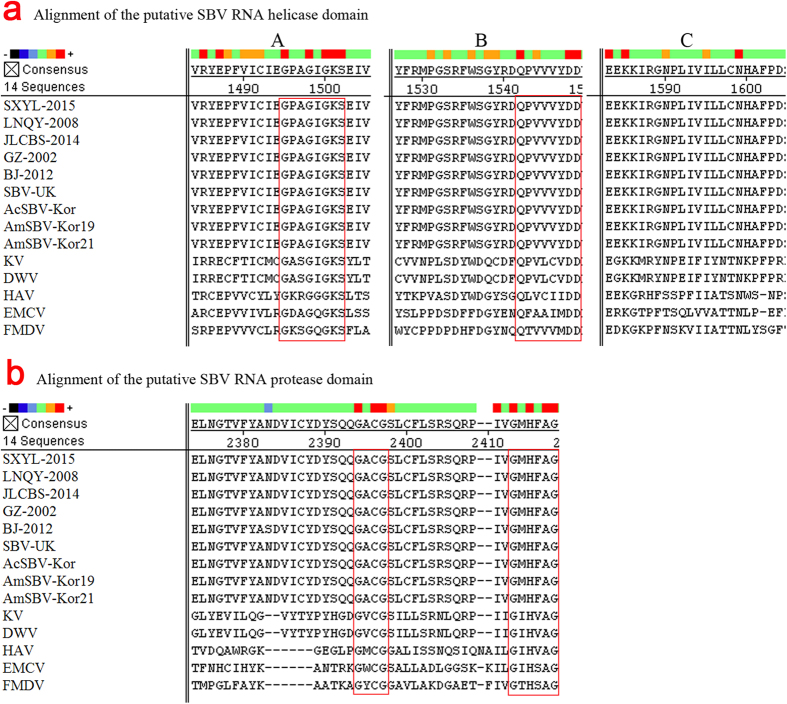
Alignment of the putative RNA helicase and protease domains. The highly conserved GxxGxGKS and Qx5DD motifs were found in helicase domains A and B, respectively, but the C domain appears to be the least conserved, containing only three of the six residues potentially associated with this site. The conserved cysteine protease motif GxCG and the putative substrate-binding residues in the GxHxxG domains were found between amino acid positions 2229 and 2288 in LNQY-2008, SXYL-2015, and JLCBS-2014.

The deduced amino acid sequences of mammalian picornaviruses and insect picornalike viruses were then aligned and compared. The results revealed that the structural proteins are located at the 5′ end and the non-structural proteins at the 3′ end^16^. The helicase domains A, B, and C^17^ are located between amino acid positions 1353 and 1490 in LNQY-2008, SXYL-2015, and JLCBS-2014. This region includes highly conserved amino acids within the first two domains, GxxGxGKS and Qx5DD in domains A and B; however, the C domain appears to be the least conserved, containing only three of the six residues potentially associated with this site (Fig. 2). The equivalent of the conserved cysteine protease motif GxCG and the putative substrate-binding residues in the GxHxxG domains were identified within the protease domains in the deduced amino acid sequences of the viruses^18^. These motifs were found between amino acid positions 2229 and 2288 (Fig. 2) in LNQY-2008, SXYL-2015, and JLCBS-2014. Similar results were obtained for foot-and-mouth disease (FMDV, GenBank: AY333431.1), hepatitis A (HAV, AB279735), encephalomyocarditis (EMCV, M81861.1), Kakugo (KV, AB070959), and deformed wing (DWV, AJ489744) viruses, between amino acid positions 1206 and 1580.

**Figure 3:**
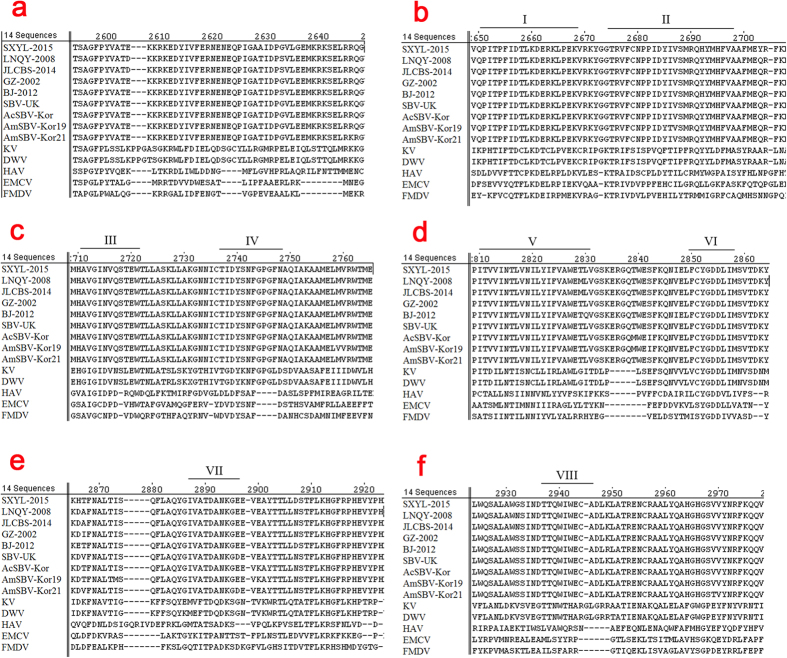
Alignment of the amino acid sequence of the RdRp of CSBV. Eight conserved domains identified in RdRp were found between amino acid positions 2444 and 2830 in LNQY-2008, SXYL-2015, and JLCBS-2014, and the motifs previously identified in RdRp are labelled I–VIII.

The amino acid sequence of the C-terminal region of CSBV polyprotein is similar to that of RdRp (RNA-dependent RNA polymerase) viruses of the Picornaviridae family (Fig. 3). Eight conserved domains identified in RdRp^17^ were also found between amino acid positions 2444 and 2830 in LNQY-2008, SXYL-2015, and JLCBS-2014.

**Figure 4:**
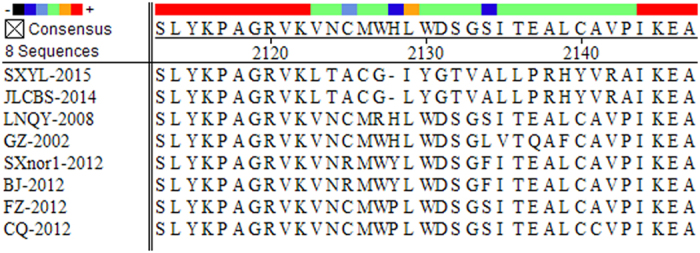
Alignment of the amino acid region 2112-2148 of non-structural proteins region of CSBV. SXYL-2015 and JLCBS-2014 have a deletion at amino acid position 2128, but LNQY-2008 does not.

Next, we determined the amino acid sequence homology among the SBV strains. Our results (Table 2, Row 4–6) showed that LNQY-2008, SXYL-2015, and JLCBS-2014 share 94.1–96.3%, 95.2–97.8%, and 94.8–97.6% sequence identity, respectively, with the other SBV isolates. In addition, SXYL-2015 and JLCBS-2014 have a deletion at amino acid position 2128, but LNQY-2008 does not (Fig. 4).

**Figure 5:**
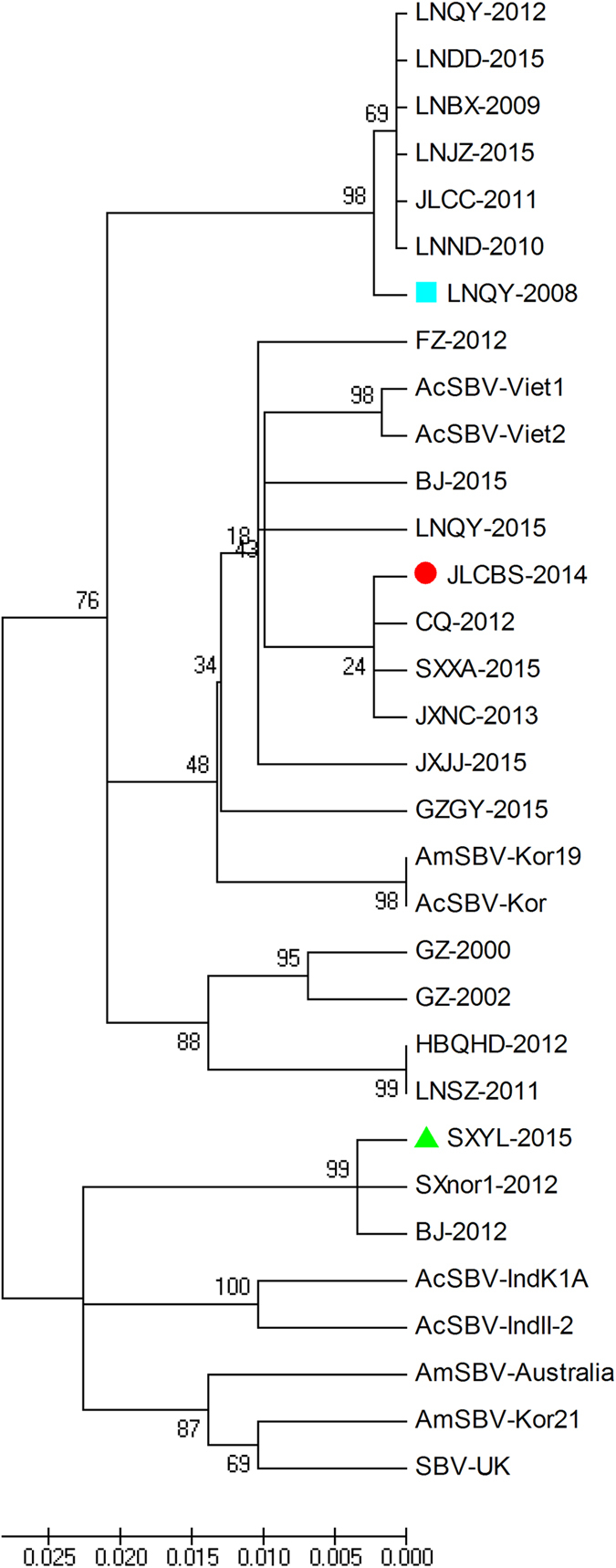
Phylogenetic analysis of the VP1 region amino acid sequences obtained from China, Korea, Vietnam, India, Australia, and the United Kingdom.

A phylogenetic tree was constructed on the basis of the high sequence variability among the partial amino acid sequences of the VP1 region obtained from China, Korea, Vietnam, India, Astralia and the United Kingdom to illustrate the probable genetic relations among the selected SBV strains. Phylogenetic analysis showed that group III and the strains isolated in Korea (AcSBV-Kor and AmSBV-Kor19, GenBank: JQ390592) and Vietnam (AcSBV-Viet1, and AcSBV-Viet2, GenBank: KM884991) could be classified into a clade. Group II was clustered into a separate subgroup except for FZ-2012. SXYL-2015, SXnor1-2012, and BJ-2012 were clustered into a subgroup, and HBQHD-2012, LNSZ-2011, GZ-2000, and GZ-2002 were also clustered into a separate subgroup, but the latter formed a closely related cluster with groups II and III (Fig. 5).

### Comparative analysis of pathogenicity

All the larvae after oral inoculation with CSBV (groups 1–15) used in this study were analysed by RT-PCR method, and the results showed that all of the other honeybee viruses were undetectable whereas CSBVs were detectable. All the larvae in the virus-free control (group 16) showed that common honeybee viruses were absent.

**Figure 6:**
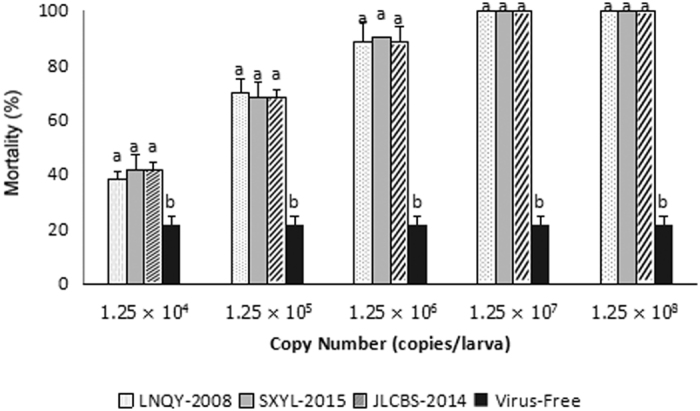
Comparative analysis of mortality rates among 2-day-old larvae infected with different dilutions of one of the three CSBV strains. There were no significant differences (P > 0.05) among the three CSBV strains when the larvae were inoculated with the same number of copies.

Two-day-old larvae were inoculated with LNQY-2008, SXYL-2015, or JLCBS-2014 (serial 10-fold dilutions), respectively, and the experiment was repeated three times. Three repeated experiments showed (Fig. 6) that the mortality rates of the larvae were 35–45%, 65–75%, and 80–95% in the groups where each larva was sequentially inoculated with 1.25 × 10^4^ copies, 1.25 × 10^5^ copies, and 1.25 × 10^6^ copies of LNQY-2008, SXYL-2015, or JLCBS-2014, respectively. In contrast, the mortality rates of the larvae were all 100% in the two groups, where each larva was sequentially inoculated with 1.25 × 10^7^ and 1.25 × 10^8^ copies of one of the three strains of CSBV. In the virus-free control groups, the mortality rates of the larvae were 20–25%. There were no significant differences in LNQY-2008, SXYL-2015, and JLCBS-2014 when the 2-day-old larvae were inoculated with the same number of copies of one of the three CSBV strains.

**Figure 7:**
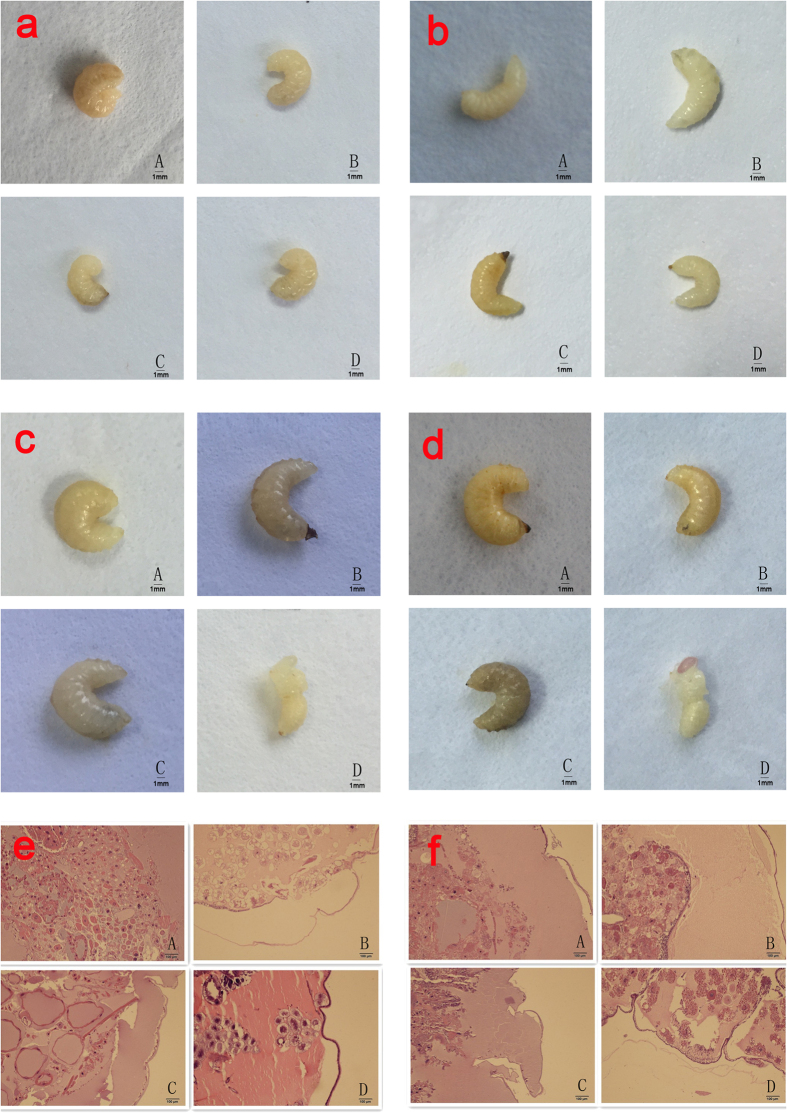
Comparative analysis of pathogenicity of the three representative strains. A, B, C, and D represent the following groups: LNQY-2008, SXYL-2015, JLCBS-2014, and Normal. (**a**, **b**, **c**, and **d**) represent larvae 2, 4, 6, and 8 days, respectively, after inoculation. (**e** and **f**) represent a histopathological slide from larvae 3 and 6 days after inoculation.

Histopathological analysis (hematoxylin and eosin [H&E] staining; Fig. 7) revealed that infection of larvae by one of the three CSBV strains caused lesions in the internal organs and tissues of the larvae. The lesions caused by each of these three strains appeared similar. Normal tissue cells after H&E staining were intact, with small round nuclei; the clearance between the epidermis and dermis was small, with few signs of granular liquid. Three days after the inoculation, histopathological analysis showed increased clearance between the epidermis and dermis, disappearance of a portion of the dermis, and deformation of the cells and nuclei. Six days after the inoculation, the gap between the epidermis and dermis increased further and was filled with a watery fluid, and became increasingly hollow. Additionally, the dermis gradually disappeared. The shapes of the cells and nuclei become irregular, and they even disintegrated in some instances. Various organelles disintegrated, resulting in cell lysis. Moreover, all the larvae inoculated with the same copy numbers of one of the three strains developed the same signs of the disease (Fig. 7). Larvae infected with one of the three CSBV strains failed to pupate, and ecdysial fluid accumulated beneath their unshed skin. Larvae changed in colour from white to pale, or even dark yellow, and died. Shortly afterwards, they dried out, forming dark brown gondola-shaped scales.

### Comparative analysis of physicochemical properties

**Figure 8:**
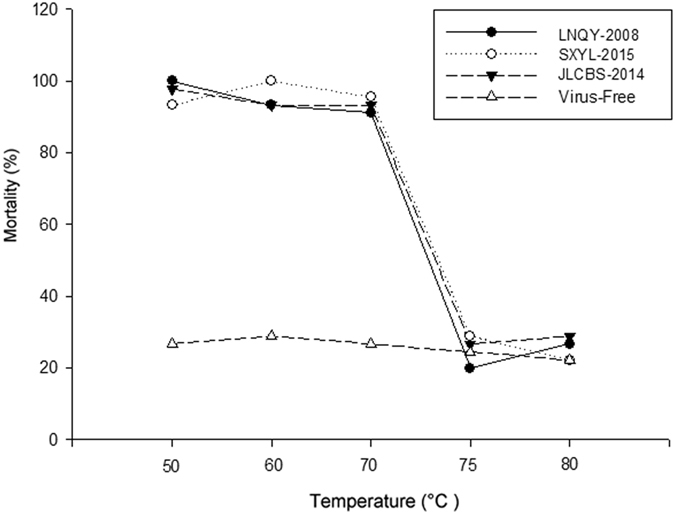
Comparative analysis of physicochemical properties of the three representative CSBV strains. Analysis of temperature resistance shows that the mortality of larvae infected by each CSBV strain pre-incubated at 50 °C, 60 °C, and 70 °C was not significantly different (P > 0.05), whereas larvae infected with the virus pre-incubated at 75 °C or 80 °C showed significantly lower mortality (P < 0.01), indicating that CSBVs can be inactivated by incubation at 75 °C for 1 h.

As shown in Fig. 8, the mortality rates of larvae infected by each CSBV strain incubated at 50 °C, 60 °C, or 70 °C were not significantly different (P > 0.05), whereas infected larvae incubated at 75 °C and 80 °C and the virus-free control showed significantly lower mortality (P < 0.01), indicating that CSBVs can be inactivated by incubation at 75 °C for 1 h. By contrast, pH 3, ethyl ether, and chloroform seemed to have no effect on viral activity because larvae infected with the viruses exposed to these conditions showed 100% mortality. Moreover, there were no significant differences in the resistance of the three CSBV strains when exposed to high temperatures, pH 3, ethyl ether, or chloroform (P > 0.05).

### Analysis of immunogenicity

**Figure 9:**
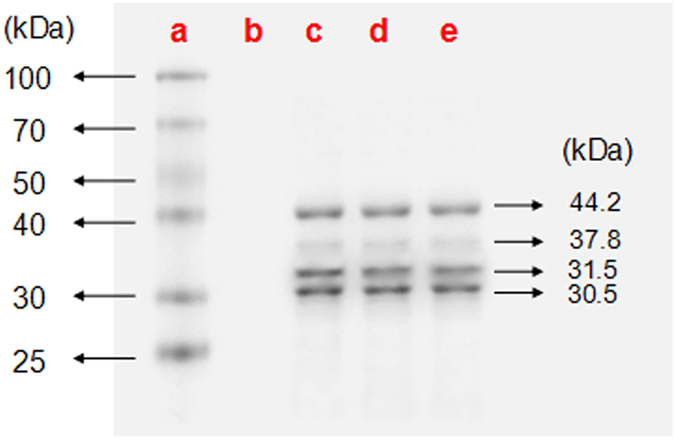
Proteins of CSBV analysed by SDS-PAGE. Proteins were resolved on 12% SDS-polyacrylamide gels following standard protocols. (**a**, **b**, **c**, **d**, and **e**) represent protein markers, virus-free control, LNQY-2008, SXYL-2015, and JLCBS-2014, respectively.

**Figure 10:**
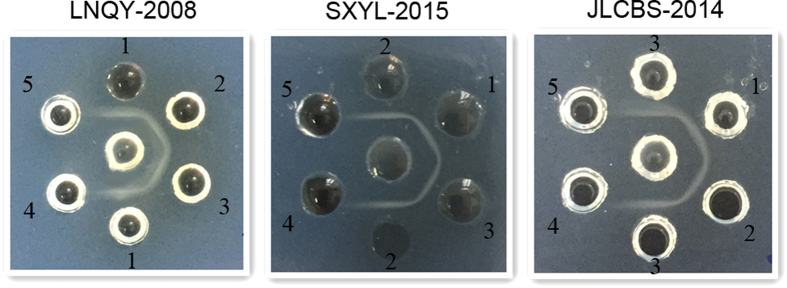
Agar gel immunodiffusion assay for LNQY-2008, SXYL-2015, JLCBS-2014. 1, 2, 3, 4, and 5 represent anti-LNQY-2008, anti-SXYL-2015, anti-JLCBS-2014, and non-immune sera and normal saline, respectively. The central holes were loaded with viral strains, and the surrounding holes were loaded with serum. Three CSBV strains yielded an immunoprecipitation line with each of the three sera but not with the non-immune serum or saline.

**Figure 11:**
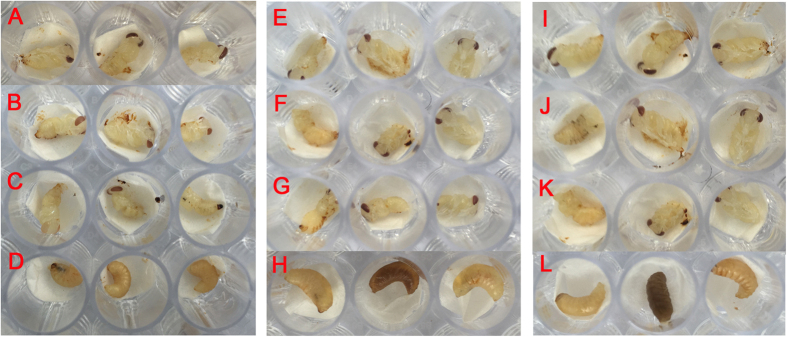
Virus neutralisation assay to examine protective immunity. Each of the three viral strains was allowed to react with each of the three antisera, to test neutralisation. In the figure, (**A–L**) represent the groups indicated below: (**A**) LNQY-2008 virus + LNQY-2008 serum, (**B**) LNQY-2008 virus + SXYL-2015 serum, (**C**) SXYL-2015 virus + JLCBS-2014 serum, (**D**) LNQY-2008 virus + non-immune serum, (**E**) SXYL-2015 virus + SXYL-2015 serum, (**F**) SXYL-2015 virus + LNQY-2008 serum, (**G**) SXYL-2015 virus + JLCBS-2014 serum, (**H**) SXYL-2015 virus + non-immune serum, (**I**) JLCBS-2014 virus + JLCBS-2014 serum, (**J**) JLCBS-2014 virus + LNQY-2008 serum, (**K**) JLCBS-2014 virus + SXYL-2015 serum, and (**L**) JLCBS-2014 virus + negative serum.

The three strains of purified CSBV have four major proteins, with estimated molecular weights of 30.5, 31.5, 37.8, and 44.2 kDa (Fig. 9). The results of agar gel immunodiffusion (AGID) assays (Fig. 10) revealed that there were three kinds of antigens and antisera, each with a clean lane. This result indicated cross-immunogenicity among the three representative strains and cross-reactivity among the three antisera, whereas the immunoprecipitation band was not observed for non-immune serum or saline. In the virus neutralisation assay (Fig. 11), the three strains of the virus were incubated with the three types of antisera and fed to healthy larvae. The larvae showed normal pupation after 4 days. No significant differences were observed among the groups (P > 0.05). Larvae inoculated with the viruses that were neutralised with non-immune serum did not show normal pupation and eventually died. Therefore, the immunisation with different CSBV strains seems to offer cross-protection.

## Discussion

The incidence of CSBV infection has increased considerably in the past few years, and the virus is seriously threatening apiculture. Currently, CSBV research is focused on genetic characterisation, cell culture, immunisation with structural proteins, and treatment^14^,^19^,^20^,^21^. Studies have shown that cross-species transmission is more frequent for RNA viruses than for other pathogens of the honeybee^22^,^23^,^24^,^25^. Since 2008, we have monitored the prevalence of CSBV in China and obtained 16 strains of CSBV from different regions and time points (Table 1). Sequence analyses of the viral *VP1* genes indicate that there are three kinds of the *VP1* gene. In this study, we compared three strains from China (LNQY-2008, SXYL-2015, and JLCBS-2014) in terms of molecular biological characteristics, physicochemical properties, pathogenicity, and immunogenicity. The nucleotide sequences of these three strains, which infect *A. cerana*, were also determined. Analysis of their molecular biological characteristics indicates that the genomes of SXYL-2015, LNQY-2008, and JLCBS-2014 contain a single, large ORF starting at nucleotide positions 177, 178, and 189, respectively, and terminating in a stop codon at nucleotide positions 8756, 8721, and 8717, respectively. Analysis of the deduced amino acid sequences of SXYL-2015, LNQY-2008, and JLCBS-2014 indicates the presence of conserved motifs within the helicase, protease, and RdRp domains, as in other viruses.

Genetic exchange (by either recombination or reassortment) plays an important role in evolution by rapidly increasing variation and was suggested to have evolved to offset fitness losses^26^. Some studies have shown that most of the genomic sequences diverged considerably in the *VP1* region^27^. In this study, we compared some of the SBV genome reported by Reddy *et al.*^27^ and that published in GenBank for our strains. We found deletions or insertions near the *VP1* gene region in structural and non-structural proteins. Our analysis revealed that amino acid deletions or insertions are common phenomena in SBV and may be associated with regional differences and host species.

The phylogenetic tree of *VP1* revealed that strains in group III and the strains isolated in Korea and Vietnam tend to be grouped together, suggesting that strains in group III might have originated in Korea in 2010 and then spread to China and Vietnam. Amino acid sequence analysis also showed less variation among group III strains. The strains in group II independently form a clade except for FZ-2012, which is closely related to group III because the mutated amino acids in FZ-2012 VP1 (C, N, V, H, Q, and A) are the same as those in strains of group III. In group I, although SXYL-2015, SXnor1–2012, and BJ-2012 were isolated from the Chinese honeybee *A. cerana*, those strains formed a closely related cluster with the strains originating overseas, such as AcSBV-IndK1A, AcSBV-IndII-2 (GenBank: JX270795), AmSBV-Australia (GenBank: KJ629183), AmSBV-Kor21, and SBV-UK. We deduced that SXYL-2015, SXnor1-2012, and BJ-2012 probably originated from SBV infecting *Apis mellifera*, and then infected *A. cerana*, indicating that SBV can cause interspecies infections, and these data are consistent with Gong’s results^28^. By contrast, HBQHD-2012, LNSZ-2011, GZ-2000, and GZ-2002 form a closely related cluster with groups II and III; this finding shows that these isolates originated from GZ-2000, which was first isolated from the Chinese honeybee *A. cerana* in Guangzhou in 2000. *VP1* variability may affect the biological characteristics of CSBV.

The high pathogenicity of CSBV towards *A. cerana* has been the focus of intensive research. By comparing different genotypes of three CSBV strains in terms of pathogenicity and the pathological damage to *A. cerana*, we found that the mortality rate of 2-day-old *A. cerana* inoculated with one of the three CSBV strains rises with the increasing CSBV copy number. When the number of copies per larva reached 1.25 × 10^7^, the mortality rate was 100%. To compare the three viral strains in terms of characteristic clinical signs and pathological changes in bee larvae, we chose this 100% lethal minimal gradient. The three CSBV strains, when inoculated into 2-day-old *A. cerana* at the same copy number, showed no significant differences in lethality, clinical signs, or pathological changes. It should be noted that artificial breeding of bee larvae cannot ensure 100% survival, and we used only a small number of selected samples, individual differences among larvae and other possible reasons may explain why the larval mortality was not entirely consistent in the three repeated experiments. Nonetheless, all of the results showed a positive correlation within a certain range between the mortality of infected larvae and the inoculated virus copy number. In addition, the characteristic clinical signs and pathological changes in the larvae were the same. Infected larvae were examined microscopically after H&E staining and showed irregularities in the shapes of cells and nuclei after infection by one of the three strains. A liquid-filled cavity was observed between the epidermis and dermis of diseased larvae. Compared to that of uninfected larvae, the body surface of infected larvae was swollen 3–4 days after the inoculation. This result may be attributed to the large gap between the epidermis and dermis. When the infection progressed, we observed disintegrated cells and various broken organelles. This finding is consistent with the typical clinical signs observed, including swelling of the larval surface and increased accumulation of cyst fluid. Furthermore, because all honeybee viruses, including CSBV, have no suitable culture system, we could not accurately measure the 50% lethal dose of CSBV in this study. Therefore, we did not carry out more in-depth and meticulous research on the entire course of the disease and conducted only a preliminary study on CSBV pathogenicity, using the number of virus copies as a quantitation standard. In the future, we will further explore the methods for determination of the 50% lethal dose, to carry out a more in-depth comprehensive comparative study on the pathogenesis of CSBV infection.

Comparative analysis of the physicochemical properties of the three strains of CSBV yielded no significant differences in sensitivity to temperature, low pH (3.0), ethyl ether, and chloroform (P > 0.05). This finding indicates that CSBVs are non-enveloped viruses that are resistant to ether and chloroform. Larvae infected by CSBV that was exposed to ethyl ether and chloroform were unable to pupate. The same results were obtained upon exposure of the viruses to pH 3, indicating that these virus strains are not inactivated by an acid. After virus inactivation at 75 °C for 1 h, the viral titre and infectivity decreased, and the inoculated larvae could then morph into pupae. In this study, we also found that a few larvae could morph into pupae after inoculation with a lethal dose of CSBVs (1.25 × 10^7^ copies/larva) that were incubated at 50 °C, 60 °C, or 70 °C. The mortality rate did not reach 100%. We presumed that biological activity of CSBV probably was affected with the increase in temperature from 50 °C to 75 °C. In addition, because of the difficulty with rearing of larvae, we could not avoid deaths of the larvae caused by differences in individual, indoor, and outdoor environments and in nutrient composition, machine operation, or other reasons; thus, ~25% of the larvae are expected to die of natural causes. To detect the death of the larvae caused by infection with CSBV preincubated at 75 °C or 80 °C, we performed a regression test on dead larvae, and it showed that there is no significant difference in the mortality rate between the reseeded group and virus-free control group (P > 0.05). These results can serve as a reference for further research on physicochemical characteristics of this virus.

SDS-PAGE analysis of CSBVs purified by caesium chloride gradient centrifugation revealed four proteins with estimated molecular weights of 30.5, 31.5, 37.8, and 44.2 kDa, in agreement with reports that the CSBV structural protein is composed of four proteins with molecular weights 30.5, 31.5, 37.8, and 44.2 kDa^12^,^29^. This finding indicates that we obtained purified viral proteins, and thus avoided contamination with bee proteins in the immunological experiments. The AGID assay is a rapid method that can be performed in a veterinary diagnostic laboratory^30^. Clear precipitation bands were obtained for combinations of each of the three viral antigens and three kinds of antisera, whereas immunoprecipitation was not detected with non-immune serum and saline control. These data provide preliminary evidence of immune cross-reactivity among these three CSBV strains and the three kinds of antisera. The virus neutralisation assay supported this hypothesis. We carried out the neutralisation reaction with a custom-made antiserum and three strains of the virus, respectively. The larvae pupated normally after inoculation by feeding. Conversely, after inoculation with the virus incubated with non-immune serum, the bee larvae did not pupate. These results further corroborate the cross-protection after immunisation with different strains of CSBV and provide experimental evidence for the production of vaccines using any of these three CSBV strains because all three can confer protective immunity against CSBV.

In this study, we found that although there are sequence-specific features in each of the three genotypes, there are no significant differences in pathogenicity, physicochemical properties, or immunogenicity. Our results lay the foundation for more in-depth research on the properties of CSBV, including epidemic patterns, mechanisms of infection, and preventive measures. The specific function of the high-variability protein structure is a good topic for future studies.

## Materials and Methods

### Sample collection

A total of 185 *A. cerana* larval samples (each collected sample included five larvae from a single colony) were obtained in China in 2008–2015 (Table 1). In all larvae, CSBV infection was detected by reverse-transcriptase PCR (RT-PCR)^31^ with VP1 primers (F: 5′-GCGGATCCATGGATAAACCGAAGGATATAAG-3′, R: 5′-GCAAGCTTTTATTGTACGCGCGGTAAATA-3′). The infection rate in a test-positive sample was calculated by means of the following formula: infection rate (%) = (total number of infected larvae ÷ total number of larvae in test-positive sample) × 100.

We selected one test-positive larva randomly for sequencing from the same colony at the same time.

### Screening representative strains by multiple sequence alignments of the *VP1* gene

Using *VP1* as a target gene, we carried out multiple gene sequence comparisons for all the CSBV isolates and reference strains in GenBank using the Clustal W method in the MegAlign software (DNA STAR, Inc., Madison, WI, USA). We named the samples after the strain that originated in the same region and showed 100% homology, then submitted the data to GenBank (CSBV uniformly renamed, first isolated geographic location + years). According to the sequence comparison results, we selected LNQY-2008, SXYL-2015, and JLCBS-2014 as representative strains.

### Isolation and purification of the representative strains

For each of the three strains (LNQY-2008: ~70.0% proportion of the infected larvae, SXYL-2015: ~80.0% proportion of the infected larvae, and JLCBS-2014: ~73.3% proportion of the infected larvae), 50 infected *A. cerana* larvae were collected. After weighing, larvae were completely homogenised in sterile water (1.5-fold amount, by weight) using a pestle and mortar. CSBV purification was performed by cesium chloride gradient centrifugation, according to Ma’s method^12^,^32^. The supernatant was then passed through a 0.45-μm cell filter first and then through a 0.22-μm cell filter. The filtrate was then fed to the second instar of *A. cerana* larvae for virus passage, and 50 diseased larvae were taken from each culture plate after 8 days. The virus was isolated and purified again by the above method. Next, virus suspension was analyzed by the RT-PCR method^31^ for the following viruses: black queen cell virus (BQCV)^33^, acute bee paralysis virus (ABPV)^33^, chronic bee paralysis virus (CBPV)^34^, deformed wing virus (DWV)^34^, kashmir bee virus (KBV)^35^, Israeli acute paralysis virus (IAPV)^36^, and CSBV^31^. Three virus suspensions, after we proved that they did not contain other viruses in addition to CSBV, were stored at −80 °C until use.

### Analysis of molecular biological characteristics

RNA was extracted from each purified virus suspension of the three CSBV strains (LNQY-2008, SXYL-2015, and JLCBS-2014), using TRIzol LS (Invitrogen, USA) and retrotranscribed into cDNA using AMV reverse transcriptase, random oligonucleotides, and oligo (dT) as primers^31^.

As previously described^12^, we designed the primers in this work on the basis of the nucleotide sequences of GZ-2002 and SBV-UK. These primers were used to prepare full-length, single-stranded cDNA of LNQY-2008, SXYL-2015, and JLCBS-2014 genomes. The cDNAs were amplified by PCR (30 cycles, annealing at 50–55 °C for 45 s, and elongation at 72 °C for 50–60 s). The 3′ end was cloned by the 3′-RACE method (Clontech). The PCR-amplified cDNAs were cloned into the pMD 18-T vector (Takara Biotechnology Co., Ltd., Dalian, China). The plasmids were then used to transform *Escherichia coli* DH5α cells (Takara Biotechnology Co., Ltd.). The plasmids were extracted using the Plasmid Extraction Kit (Axygen Biotechnology Co., Ltd.).

Nucleotide sequencing was performed by Sangon Biotech Co., Ltd. The nucleotide sequences from all of the fragments were assembled to build a continuous complete sequence by means of the DNASTAR software. Sequence analysis was performed by the Clustal W method in the MegAlign software. Phylogenetic trees were constructed by the neighbour-joining method (*p* = distances), and up to 1000 bootstrapping replicates were used in the MEGA 5.0 software for *VP1* region amino acid sequences obtained from China, Korea, Vietnam, India, Australia, and the United Kingdom.

### Comparative analysis of pathogenicity

As described previously^37^, PCR probes were used to measure the copy numbers of LNQY-2008, SXYL-2015, and JLCBS-2014. Three CSBV strains were subjected to 10-fold serial dilutions. The amounts of the virus after dilution were 6.24 × 10^2^, 6.24 × 10^3^, 6.24 × 10^4^, 6.24 × 10^5^, and 6.24 × 10^6^ copies/μL.

Two-day-old *A. cerana* larvae from a single mated honeybee queen in a healthy apiary in Jinzhou, Liaoning Province, were orally inoculated with CSBV. To obtain age controlled larvae, the queen was caged on a comb after fumigation and disinfection, and left to lay eggs for 6 h. Twenty hours after larval eclosion or 92 h after oviposition, the comb containing 2-day-old larvae was retrieved from the colony. A total of 320 larvae were selected and distributed into 16 groups randomly, each group contained 20 larvae, groups 1, 4, 7, 10, and 13 were inoculated with LNQY-2008 at 1.25 × 10^4^, 1.25 × 10^5^, 1.25 × 10^6^, 1.25 × 10^7^, and 1.25 × 10^8^ copies/larva, respectively; groups 2, 5, 8, 11, and 14 were inoculated with SXYL-2015 at 1.25 × 10^4^, 1.25 × 10^5^, 1.25 × 10^6^, 1.25 × 10^7^, and 1.25 × 10^8^ copies/larva, respectively; groups 3, 6, 9, 12, and 15 were inoculated with JLCBS-2014 at 1.25 × 10^4^, 1.25 × 10^5^, 1.25 × 10^6^, 1.25 × 10^7^, and 1.25 × 10^8^ copies/larva, respectively; and group 16 served as a virus-free control. The above virus inoculation assay was performed twice after the end of the first experiment.

Each larva was fed 20 μL of a virus suspension mixed with an equal amount of basic larval diet^38^ (BLD; 50% royal jelly, 37% sterile water, 6% glucose, 6% fructose, 1% yeast extract) at 95% relative humidity and 34 °C. The virus-free control was fed 20 μL of sterile water with an equal amount of BLD. BLD was used subsequently for daily feeding. The clinical signs in each group of larvae were examined and recorded every day until death of larvae. All the larvae that were orally inoculated in this study were analysed by the RT-PCR method^31^ for BQCV^33^, ABPV^33^, CBPV^34^, DWV^34^, KBV^35^, IAPV^36^, and CSBV^31^.

Infected larvae were selected 1–6 days post-infection for histopathological examination of tissue sections. The experiment included formalin-fixed paraffin-embedded (FFPE) diseased larvae, which were infected by LNQY-2008, SXYL-2015, or JLCBS-2014. H&E-stained paraffin-embedded sections obtained from larvae were examined by light microscopy.

### Comparative analysis of physicochemical properties

According to the conventional method^39^, equal amounts of each of the three virus suspensions and sterile water were individually incubated at varying temperatures (50 °C, 60 °C, 70 °C, 75 °C, or 80 °C) in a water bath for 1 h to carry out the temperature resistance experiment, and each group contained 15 larvae. Purified virus suspensions (pH adjusted to 3.0 using an HCl solution, 0.1 M, pH 3.0) were incubated at a constant temperature of 37 °C in a water tank for 12 h, followed by adjusting the pH of the solution to 7.2 with a 5.6% NaHCO_3_ solution. Next, sensitivity to pH 3.0 was measured. To analyse sensitivity to chloroform, pure chloroform was added to purified virus (at a final concentration of 4.8%); the mixture was rocked gently at 4 °C for 10 min and centrifuged at 500 rpm for 5 min. The supernatant was extracted to analyse the viral activity. To analyse sensitivity to ethyl ether, each virus suspension was mixed with ethyl ether, and placed on an oscillator shocking for 60 min, and centrifuged at 2000 rpm for 20 min. We used a capillary with appropriate percussion and absorbed the virus for ether sensitivity test. Then, according to the above method, a 2-day-old *A. cerana* larva was fed 20 μL of the virus suspension (1.25 × 10^7^ copies) along with an equal amount of BLD, and was examined after 6 days to determine whether the larvae developed the signs of CSD (to detect CSBV infection). The virus-free control larvae were fed 20 μL of sterile water with an equal amount of BLD. A total of four treatments (replicated thrice) were carried out.

### Analysis of immunogenicity

Four- to 6-week-old female pathogen-free BALB/c mice were randomly subdivided into four groups with five mice in each group: negative control, LNQY-2008, SXYL-2015, and JLCBS-2014. All animal procedures were approved by the Ethics Committee of Liaoning Medical University. Immunisations were performed via three intraperitoneal injections at 15-day intervals. All antigens were emulsified with complete Freund’s adjuvant for the first immunisation and with incomplete Freund’s adjuvant for subsequent immunisations; PBS served as a negative control. The amount of each antigen used for each immunisation was 20 μg/mouse. The specific antibodies in the serum samples were detected by an enzyme-linked immunosorbent assay (ELISA) and the absorbance at 490 nm was measured on an ELISA plate reader^40^. Serum samples were separated from blood by centrifugation at 3600 rpm for 15 min and then stored frozen until use. Three kinds of CSBV proteins were separated by SDS-PAGE. The proteins were resolved on 12% SDS-polyacrylamide gels using standard protocols^41^.

One gram of agarose and 8 g of NaCl were added to 100 mL of phosphate buffer (0.01 M, pH 7.2); the mixture was shaken well, and microwaved for 2 min to prepare an agar solution, which was slightly cooled and poured into Petri dishes (90 mm in diameter; 20–22 mL of agar per plate) and were allowed to solidify. Seven wells were made in the agar plates for each experimental group; the central hole was loaded with one of the three CSBV strains, and the surrounding wells were loaded with antisera against LNQY-2008, SXYL-2015, or JLCBS-2014 or non-immune serum. The AGID assay was performed for 24–48 h at 37 °C in a humidified chamber, and the results were examined thereafter.

Anti-LNQY-2008, anti-SXYL-2015, anti-JLCBS-2014, and non-immune sera were inactivated at 56 °C for 30 min and diluted 1:10; 15 μL of each serum was allowed to react with 15 μL of a LNQY-2008, SXYL-2015, or JLCBS-2014 suspension (9.36 × 10^6^ copies/μL) at 37 °C for 1 h and then were mixed with 15 μL BLD. The mixture was fed to 2-day-old instar larvae. Pupal development and mortality were examined and recorded.

### Statistical Analysis

Data on the mortality rates expressed as percentages were normalised using arcsine square root transformation and were subjected to repeated-measures analysis of variance with the Bonferroni adjusted *post hoc* pair test, in the SPSS 17.0 software (SPSS Inc., Chicago, IL, USA). If Mauchly’s test of sphericity showed no significant differences in the repeated-measures data (P > 0.05), then normal one-way analysis of variance was performed. Differences were considered significant at P < 0.05.

## Additional Information

**How to cite this article**: Hu, Y. *et al.* A comparison of biological characteristics of three strains of Chinese sacbrood virus in *Apis cerana. Sci. Rep.* **6**, 37424; doi: 10.1038/srep37424 (2016).

**Publisher’s note:** Springer Nature remains neutral with regard to jurisdictional claims in published maps and institutional affiliations.

## Figures and Tables

**Table 1:** The *A. cerana* larval samples and infection rates (%) in test-positive samples calculated in China in 2008–2015.

No.	Isolate	Geographic location	Year	Positive sample/Total sample	Total number of infected larvae	Infection rate (%)	Accession No.
a	JLCBS-2014	Changbaishan, Jilin	2014	3/5	11	73.3	KU574661
			2015	2/5	7	70.0	
b	SXXA-2015	Xian, Shanxi	2015	2/3	5	50.0	KX254338
c	BJ-2015	Beijing	2015	4/6	13	65.0	KX254340
d	LNQY-2015	Qingyuan, Liaoning	2015	2/3	6	60.0	KX254337
e	GZGY-2015	Guiyang, Guizhou	2015	3/5	10	66.7	KX254332
f	JXJJ-2015	Jiujiang, Jiangxi	2015	3/5	11	73.3	KX254333
g	LNSZ-2011	Suizhong, Liaoning	2011	2/4	6	60.0	JX854441
			2012	0/3	0	0	
			2013	0/3	0	0	
			2014	0/4	0	0	
			2015	0/3	0	0	
h	HBQHD-2012	Qinhuangdao, Heibei	2012	2/3	7	70.0	JX854436
			2013	0/3	0	0	
			2014	0/3	0	0	
			2015	0/4	0	0	
i	SXYL-2015	Yulin, Shanxi	2015	3/4	12	80.0	KU574662
j	LNQY-2008	Qingyuan, Liaoning	2008	4/6	14	70.0	HM237361
			2009	4/5	13	65.0	
			2010	3/4	9	60.0	
			2011	0/4	0	0	
		Siping, Jilin	2010	3/5	9	60.0	
		Tieling, Liaoning	2010	5/5	16	64.0	
k	LNBX-2009	Benxi, Liaoning	2009	4/5	13	65.0	JX854438
			2010	2/5	6	60.0	
			2011	0/3	0	0	
			2012	0/3	0	0	
			2013	0/4	0	0	
			2014	3/5	8	53.3	
			2015	0/4	0	0	
l	JLCC-2011	Changchun, Jilin	2011	2/4	6	60.0	JX854437
			2012	4/4	13	65.0	
			2013	0/3	0	0	
			2014	0/5	0	0	
			2015	0/4	0	0	
m	LNND-2011	Nandian, Liaoning	2011	2/3	6	60.0	JX854439
			2012	0/3	0	0	
			2013	0/3	0	0	
			2014	0/5	0	0	
			2015	0/3	0	0	
n	LNQY-2012	Qingyuan, Liaoning	2012	3/5	9	60.0	JX854440
			2013	3/4	10	66.7	
			2014	2/5	5	50.0	
o	LNDD-2015	Dandong, Liaoning	2015	4/6	14	70.0	KX254334
p	LNJZ-2015	Jinzhou, Liaoning	2015	2/4	5	50.0	KX254336

^*^Each collected sample included five larvae from a single colony. Infection rate (%) = (total number of infected larvae ÷ total number of larvae in test-positive sample) × 100.

**Table 2:** Nucleotide and deduced amino acid sequences homology (%) among the three CSBV representative strains and the reference sequences.

	GZ-2002	BJ-2012	FZ-2012	SXnor1-2012	SBV-UK	AcSBV-Kor	AmSBV-Kor21	AcSBV-IndK1A	AcSBV-Viet1
LNQY-2008	93.7%	93.4%	93.7%	93.4%	90.4%	92.7%	90.3%	93.2%	92.7%
SXYL-2015	93.7%	96.5%	93.2%	96.9%	90.2%	92.7%	89.8%	92.5%	92.6%
JLCBS-2014	94.7%	92.7%	96.2%	92.8%	90.0%	94.0%	89.9%	92.6%	95.1%
LNQY-2008	95.8%	95.8%	96.3%	96.2%	94.9%	95.0%	94.3%	94.1%	95.3%
SXYL-2015	95.8%	97.2%	95.6%	97.8%	96.0%	96.6%	95.3%	95.2%	96.3%
JLCBS-2014	96.3%	95.2%	97.2%	95.7%	95.8%	97.3%	95.1%	94.8%	97.6%

Homology (%) of the deduced amino acid sequences for the coding regions among the three CSBV representative strains and the reference sequences.
